# Managing Arrhythmias in Cardiogenic Shock: Insights Into Milrinone and Dobutamine Therapy

**DOI:** 10.7759/cureus.76089

**Published:** 2024-12-20

**Authors:** Jodi-Ann A Fletcher, Savitri Poornima Halaharvi, Cinda Manuvel, Alexander L Brooks, Randev A Wannakuwatte, Eugenio Lucano Gomez, Stacy Ann Reid, Nithin Karnan, Snehitha Reddy, Shriya Maini, Bhargav A Said, Zahra Nazir

**Affiliations:** 1 Internal Medicine, St. George's University School of Medicine, St. George, GRD; 2 Internal Medicine, Rajiv Gandhi University of Health Sciences, Bengaluru, IND; 3 Internal Medicine, Believers Church Medical College, Kuttapuzha, IND; 4 Internal Medicine and Primary Care, Ivy Green Medical, Kingston, JAM; 5 Surgery, University of Colombo, Colombo, LKA; 6 Medicine and Surgery, Universidad Autonoma de Nuevo Leon, San Nicolás de los Garza, MEX; 7 Medicine and Surgery, The University of the West Indies, Kingston, JAM; 8 Internal Medicine, K.A.P. Viswanatham Government Medical College, Tiruchirappalli, IND; 9 Medicine, Zhengzhou University, Zhengzhou, CHN; 10 Internal Medicine, Dayanand Medical College and Hospital, Ludhiana, IND; 11 Internal Medicine, University of Visayas - Gullas College of Medicine, Cebu City, PHL; 12 Internal Medicine, Combined Military Hospital, Quetta, PAK

**Keywords:** cardiogenic shock, dobutamine, heart failure, milrinone, vasopressor

## Abstract

Shock is a state of inadequate perfusion that affects vital organs. Cardiogenic shock (CS) predisposes patients to various arrhythmias. The adverse effect depends on intervention and pharmacogenomics. This narrative review sheds light on treatment strategies for arrhythmias caused by milrinone and dobutamine when managing CS. Dobutamine, through beta-1 agonism, and milrinone, by phosphodiesterase-3 inhibition, increase cardiac contractility by enhancing the availability of calcium to the myocardium. Dobutamine is also a beta-2 agonist, and milrinone is a phosphodiesterase-3 inhibitor; both result in peripheral vasodilation, leading to their use preferentially in patients with CS with normotensive blood pressure. To narrow down relevant literature, various electronic databases, including PubMed, Google Scholar, and Cochrane Library, were searched. The review revealed limited evidence favoring either milrinone or dobutamine as the preferred inotropic agent for managing CS, but it did reveal that though hospital stays using dobutamine were shorter, mortality from its induced arrhythmias led to an increase in all-cause mortality rates. Both proarrhythmic agents triggered ventricular and supraventricular tachyarrhythmias, some requiring cardioversion while others are non-sustained and managed medically or symptomatically. Though neither agent has a specific reversal agent, the effect of dobutamine was seen to be successfully aborted using intravenous ultrashort half-life beta-blockers (such as esmolol). The findings accentuated the critical need for a tailored approach to managing these iatrogenic arrhythmias, emphasizing clinical vigilance and individualized patient care.

## Introduction and background

Approximately 40,000 to 50,000 people are affected by cardiogenic shock (CS) in the United States of America every year, with a mortality rate of 50% [1]. CS is when the end organs are hypoperfused, resulting in reduced cardiac output and decreased oxygen supply. It is a consequence of many heart diseases like acute heart failure, severe valvular diseases, and acute myocardial infarction [2]. CS is associated with symptoms such as acute shortness of breath, tachycardia, loss of consciousness, and hypotension. In the setting of acute myocardial infarction-induced CS, symptoms may include squeezing chest pain, sweating, and lightheadedness. It is a life-threatening condition and requires hospitalization. CS often has a high mortality of 40% [3,4]. Inotropes, most commonly dobutamine and milrinone, are used to manage CS by increasing the contractility of the myocardium under close monitoring [5-10]. Despite their regular use, several studies have shown that these inotropes are associated with arrhythmias in CS, and no standardized guidelines currently exist for managing these arrhythmias [5,7-13]. This narrative review aims to highlight current management strategies for arrhythmias in CS patients treated with milrinone and dobutamine.

## Review

**Figure 1:**
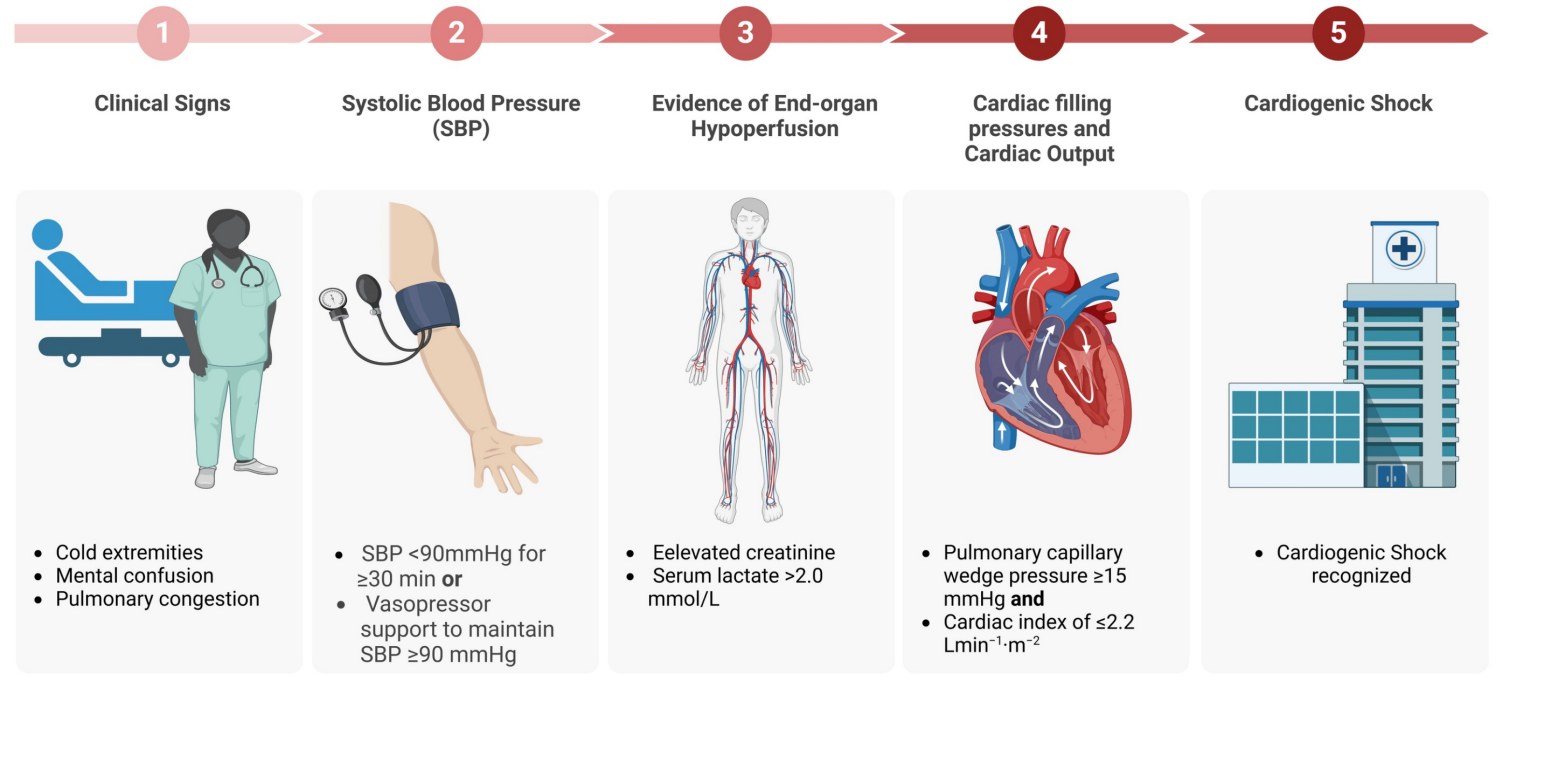
Proposed parameters for the clinical diagnosis of cardiogenic shock Image Credit: Jodi-Ann A SBP: systolic blood pressure

CS is critical cardiac dysfunction where the heart fails to maintain adequate perfusion pressure to end organs, resulting in clinical and biochemical evidence of tissue hypoperfusion. Historically, the definition of CS has varied across clinical trials, as depicted in Figure 1.

However, commonly included criteria include systolic blood pressure (SBP) <90 mmHg for ≥30 min or vasopressor support to maintain SBP ≥90 mmHg, end-organ hypoperfusion with elevated creatinine, serum lactate >2.0 mmol/L, and clinical signs such as cold extremities, mental confusion, and pulmonary congestion [14,15]. The shock trial additionally includes elevated cardiac filling pressures measured by pulmonary capillary wedge pressure (PCWP) ≥15 mmHg and low cardiac output indicated by a cardiac index of ≤2.2 L/min/m^2^ [16].

Acute coronary syndrome (ACS) presenting with myocardial infarction is responsible for most cases of CS (81%), as reported in the CardShock study [17]. In comparison, acute decompensated heart failure (ADHF) is the second most common cause of CS, resulting in almost 30% of cases as investigated by Kar et al. [18]. Advanced valvular heart disease and prosthetic dysfunction, arrhythmias, myocarditis, stress-induced cardiomyopathy, peripartum cardiomyopathy, acute coronary dissection, and thyroid disorders are among the other conditions that may cause CS [19-22].

**Figure 2:**
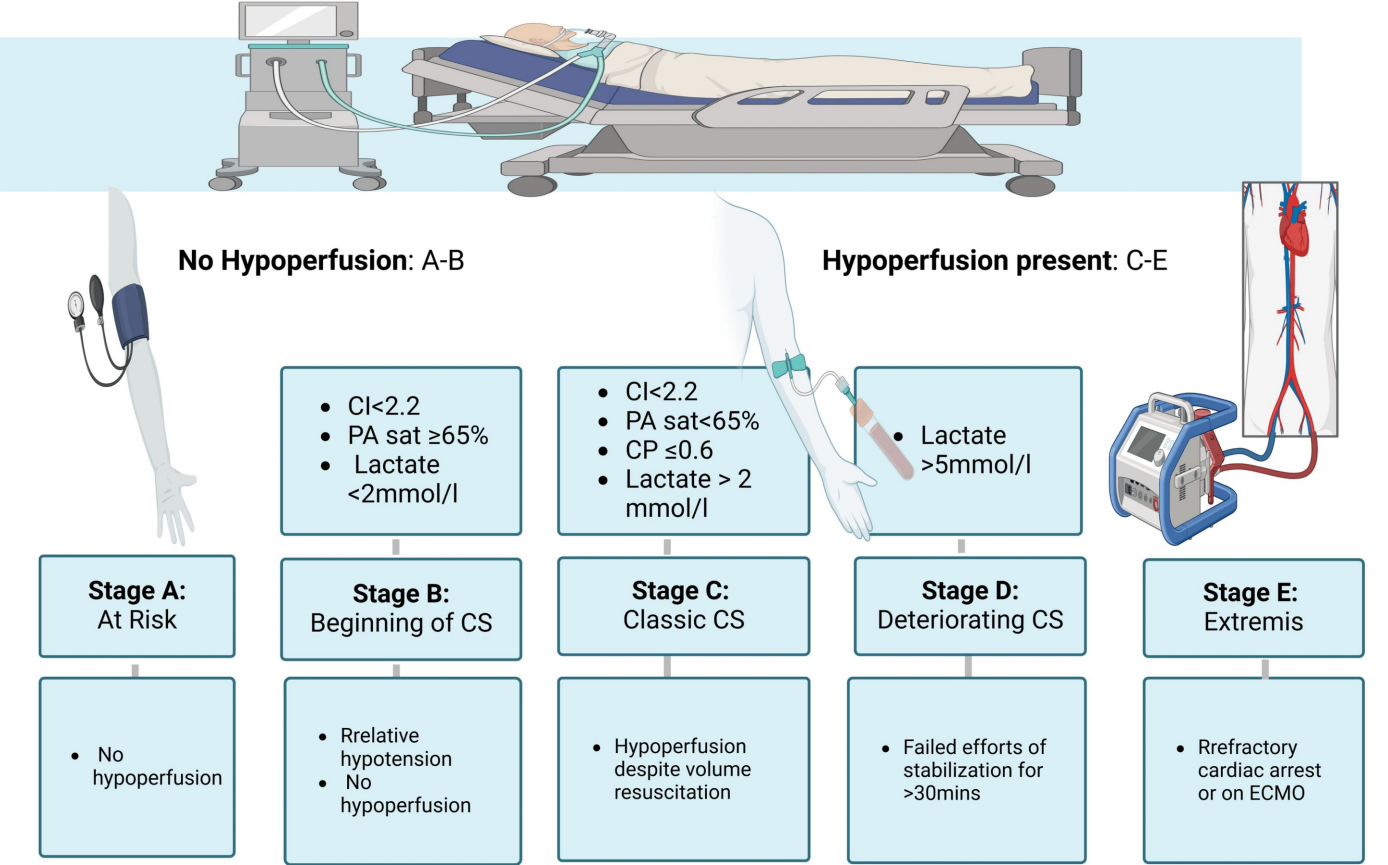
Society for Cardiovascular Angiography and Interventions (SCAI) classification of cardiogenic shock Image Credit: Jodi-Ann A CS: cardiogenic shock; ECMO: extracorporeal membrane oxygenation; PA: pulmonary artery; CI: cardiac index; CP: cardiac power

CS can be classified based on the patient's clinical status and hemodynamic parameters. Clinically, according to the classification presented by the Society for Cardiovascular Angiography and Interventions (SCAI), there are five stages, as shown in Figure 2, A describes being at risk of CS with no hypoperfusion, B refers to the beginning of CS with relative hypotension without hypoperfusion (CI < 2.2, pulmonary artery (PA) sat ≥ 65%, lactate < 2 mmol/l), C describes the classic with hypoperfusion that requires intervention beyond volume resuscitation (CI < 2.2, PA sat < 65%, cardiac power (CP) ≤ 0.6, lactate > 2 mmol), D describes deterioration with failed efforts of stabilization for >30 mins (lactate > 5 mmol/l), E describes extremis with refractory cardiac arrest or being supported by extracorporeal membranous oxygenation facilitated cardiopulmonary resuscitation [23].

**Figure 3:**
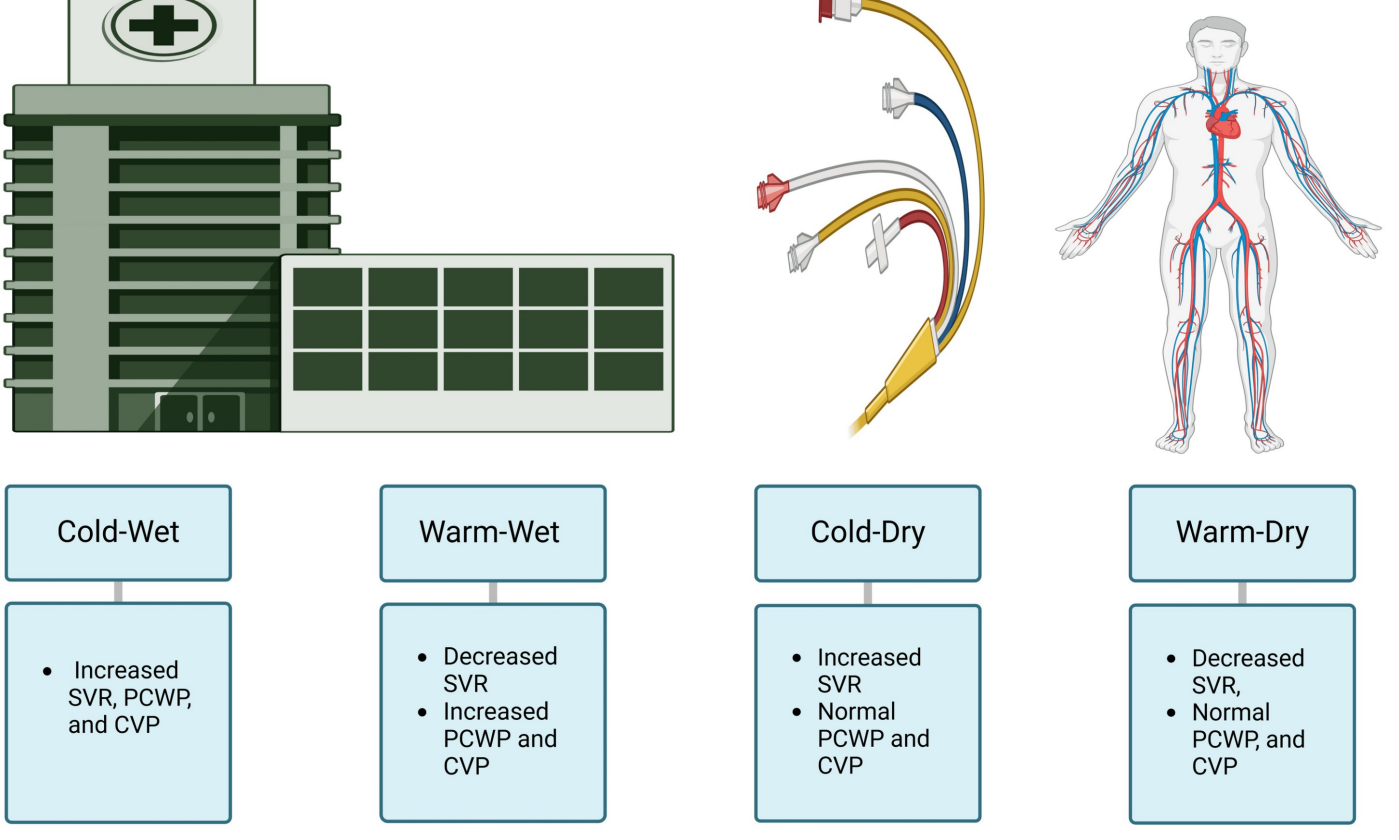
Hemodynamic presentation in cardiogenic shock Image Credit: Jodi-Ann A SVR: systemic vascular resistance; PCWP: pulmonary capillary wedge pressure; CVP: central venous pressure

Hemodynamically, CS is commonly associated with a low cardiac index; additionally, it can be classified based on systemic vascular resistance (SVR), PCWP, and central venous pressure into four categories, as shown in Figure 3.

1) "Cold-wet" (increased SVR, PCWP, and central venous pressure (CVP)) is caused by left ventricular dysfunction and represents the majority of cases; 2) "Warm-wet" (increased PCWP and CVP, decreased SVR) is associated with a systemic inflammatory response; 3) "Cold-dry" (increased SVR and normal PCWP and CVP) indicates hypoperfusion without congestion; and 4) "Warm-dry" (decreased SVR, normal PCWP, and CVP) indicates preserved perfusion to vital organs despite cardiac dysfunction due to compensatory mechanisms [24,25]. The hemodynamic classifications are essential as they help formulate treatment plans and predict the prognosis [26].

Current management strategies

Prompt recognition and targeted interventions to restore adequate tissue perfusion while mitigating further cardiac injury are vital in the management of CS. Standardized multidisciplinary team-based care is essential as CS is associated with high mortality and highly variable management strategies despite advances in care [27]. When a clinical shock state is identified with low SBP (<90 mmHg), evidence of end-organ hypoperfusion, and a lactate level of >2 mmol/L, the patient should be transferred to the cardiac ICU or the cardiac catheterization lab for further management.

The patient should undergo central venous catheterization to administer vasoactive drugs, transduce CVP, and measure central venous saturation. At the same time, comprehensive echocardiography should be performed to assess left ventricular and right ventricular function and identify the etiology of CS [28]. If acute myocardial infarction is suspected, the patient should undergo coronary angiography and revascularization [29]. Inotropes and vasopressors are essential medications used to support perfusion pressure by increasing the cardiac output and SVR to maintain SBP, as seen in most patients enrolled in the European Society of Cardiology (ESC) heart failure long-term registry [30]. Norepinephrine was the best first-line vasopressor medication, favored over epinephrine and dopamine, as in the OptimaCC trials and meta-analysis by Rui et al. [31], the inotropes dobutamine and milrinone, which are the subjects of this study, increase cardiac output to improve perfusion pressure and are integral components of the overall management strategy. However, milrinone has safety concerns due to its arrhythmogenic burden and increased myocardial oxygen demand when used in CS associated with ACS. Dobutamine is a class IIb/C recommendation in the ESC guidelines for acute and chronic failure owing to the lack of clinical data in this setting [32-34]. Milrinone is combined with vasopressin in low doses to offset its vasodilatory effects [35]. Levosimendan is another drug that is effective in patients with chronic beta-blocker therapy and pulmonary hypertension [36].

If the hemodynamic parameters are poor despite pharmacologic attempts to maintain perfusion, interventional or surgical procedures may be employed. Such strategies include percutaneous mechanical circulatory support (PMCS) as a temporary measure to buy time for recovery, the insertion of a permanent left ventricular assist device, or definitive cardiac transplantation [37]. Some PMCS systems include intra-aortic balloon pumps, Impella systems, TandemHeart, and extracorporeal membrane oxygenation (ECMO) [38]. These modalities increase the cardiac output at the cost of significant complications such as limb ischemia, bleeding, hemolysis, thrombocytopenia, septal puncture in the complex procedure of placing the TandemHeart, renal failure, and Harlequin syndrome, where incomplete retrograde filling and oxygenation causes upper body hypoxia as seen in veno-arterial ECMO. These complications highlight the advantages of effective pharmacological treatment [39].

**Figure 4:**
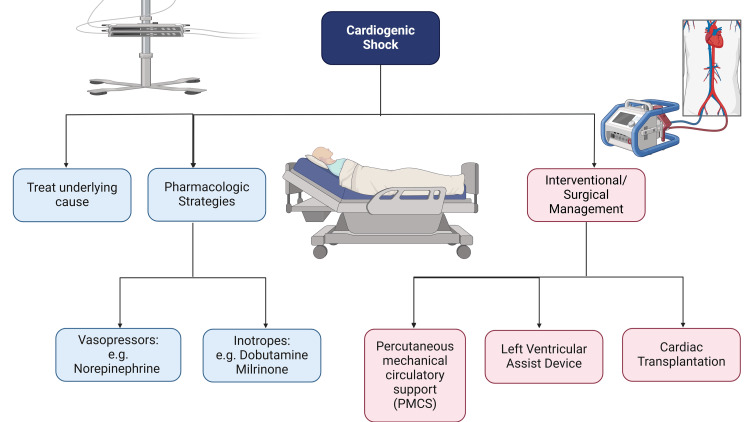
Management strategies for cardiogenic shock Image Credit: Jodi-Ann A

CS necessitates a comprehensive understanding of its characteristics and a careful approach to addressing the dynamic interplay of hemodynamic derangements, as depicted in Figure 4.

Therefore, it is paramount to consolidate the knowledge of milrinone and dobutamine to extract their maximum potential for the treatment of CS while being judicious with their use regarding the arrhythmogenic and ischemic burden on the heart.

Inotropes are routinely used for the management of CS, which is the most critical presentation of heart failure. Dobutamine and milrinone, the two most commonly used inotropes in CS, enhance calcium availability to the myocardial contractile proteins. Calcium availability to the myocardium is mediated by secondary messengers such as cyclic adenosine monophosphate (cAMP), which controls voltage-gated calcium channels through the action of various protein kinases [40]. Dobutamine binds to and triggers the beta-1 receptors in the myocardium, which then initiates the conversion of adenosine triphosphate to cAMP, leading to increased heart contractility. Increasing myocardial contractility reduces the end-systolic volume (ESV), which will increase stroke volume and help alleviate symptoms of CS [40,41]. Dobutamine is also a beta-2 agonist and causes significant peripheral vasodilation [6]. In contrast, milrinone inhibits phosphodiesterase-3, an enzyme responsible for cAMP breakdown [1]. The inhibition of phosphodiesterase-3 results in a substantial decrease in systemic and pulmonary vascular resistance, owing to the significant vasodilation it induces [8,42].

A randomized clinical trial (RCT) by Mathew et al. [43], aimed at finding the therapeutic efficacy between dobutamine and milrinone in patients above 18 years with CS, did not reveal a significant advantage of one drug over the other in terms of in-hospital death from any cause, resuscitated cardiac arrest, myocardial infarction, stroke, transient ischemic attacks, or renal replacement therapy [43]. Another systematic review and meta-analysis by Abdel-Razek et al. [2] postulated that no significant difference between the two inotropes regarding ICU length of stay or clinically significant arrhythmias was produced [2]. Fan et al. [44], after doing a meta-analysis of seven RCTs and observational studies comparing outcomes between milrinone and dobutamine, also revealed a higher incidence of all-cause mortality in dobutamine patients. However, again, there was not much difference in the length of stay in the ICU or the mean duration of hospitalization. The study did suggest that milrinone was better suited for patients with CS with concomitant pulmonary hypertension/right ventricular failure due to its ability to be a positive inotropic agent without increasing myocardial oxygen demand as well as having vasodilatory actions on pulmonary vasculature [44]. The lower mortality rate attributed to milrinone is due to improvements in hemodynamic parameters like increased stroke volume and better pulmonary artery compliance (CPA), and the higher mortality rate in dobutamine is due to its potential to increase myocardial oxygen demand and incidence of tachyarrhythmias [10,45].

Dobutamine is proarrhythmic, inducing both supraventricular tachycardias (SVTs) and ventricular arrhythmias [46,47]. Dobutamine may provoke ventricular arrhythmias by several mechanisms. Dobutamine increases myocardial consumption, leading to an increase in the risk of arrhythmia [45]. It has varying effects on action potential duration, corrected QT (QTc) interval, and QRS duration in normal and ischemic myocardium. This abnormal conduction in adjacent ischemic and non-ischemic myocardium areas creates β-receptor-mediated (reentry) arrhythmogenesis [48]. Second messengers like cAMP can increase intracellular calcium concentration, leading to increased automaticity in the ventricular myocardium. Finally, β-receptor stimulation reduces plasma potassium levels, which may temporarily predispose patients to ventricular arrhythmias. In some safety studies, ventricular arrhythmias have been related to impaired left ventricular function [48,49].

Konstam and Cody [40] documented arrhythmias associated with milrinone infused over 48 hours in patients with heart failure. Ventricular arrhythmias were seen in 12.2%, ventricular premature beats in 7.9%, and ventricular tachycardia (VT) in 4.2%, for which the only patient with sustained VT required cardioversion. Supraventricular arrhythmias were seen in 4.8% of patients. When compared to other studies, it was concluded that intravenous milrinone was proarrhythmic in up to 21% but rarely correlated to adverse clinical events in patients treated for heart failure [40].

Furthermore, in the setting of CS, both dobutamine and milrinone were associated with clinically significant arrhythmias, resulting in increased mortality. Lewis et al. [13] further established that dobutamine was associated with arrhythmias in 62.9% of patients with CS, attributed mainly to sinus tachycardia. As compared to milrinone, dobutamine was associated with more cases of arrhythmias (62.9% vs. 32.8%, p < 0.01), and arrhythmias were the most common cause of discontinuation of the drug. No statistically significant difference in the rate of atrial fibrillation between milrinone and dobutamine was observed. These arrhythmias were most often treated with either chemical or electrical cardioversion. Supraventricular arrhythmias were associated with resuscitated cardiac arrests and more prolonged in-hospital stays. However, the subset of patients with ventricular arrhythmias was associated with significantly increased resuscitated cardiac arrests and mortality [13].

Risk factors for arrhythmias caused by milrinone and dobutamine

Despite their therapeutic benefits, these drugs carry a significant risk of inducing arrhythmias, which can further complicate patient care. Recognizing the risk factors for arrhythmias associated with these medications is essential for their safe administration. This section will delve into various factors that influence the likelihood of arrhythmias, including the duration and dosage of treatment, the presence of pre-existing arrhythmias, and the patient's comorbid conditions. By understanding these risks, clinicians can better balance the efficacy and safety of milrinone and dobutamine in managing heart failure. The risk factors for the arrhythmias are as follows:

Duration of Administration

It has been observed that continuous, long-lasting management of refractory chronic heart failure with intravenous inotropes expands the chances of fatality, primarily because of their proarrhythmic effects [50]. About 28% excess mortality was seen with long-term milrinone administration in contrast to using a placebo in a clinical trial of patients with severe heart failure, and this was thought to be related to the proarrhythmic effect of the inotropes [40].

Dose of the Inotropic Agent

Dobutamine has been seen to increase the heart rate in animal models and humans in a dose-dependent fashion. Patients who are being given surplus doses of dobutamine and have an underlying arrhythmia and previously diagnosed heart failure are at higher risks of developing new arrhythmias [7].

However, ventricular electrical activity changes caused after milrinone administration are not seen to have a dose-dependent relationship [51]. Moreover, there has been a difference in risk profile with the use of oral inotropes versus intravenous lines, with more complications reported with the latter method of administration [52].

Pre-diagnosed Arrhythmias

These inotropic agents' arrhythmogenic effects are due to increased myocardial contractility, potentially negatively impacting myocardial oxygen balance, myocardial perfusion, and electrical stability [53]. Therefore, this aligns with previous reports of new arrhythmias developing following inotropic administration in patients with pre-diagnosed arrhythmias.

Comorbidities

These inotropes are connected to increased arrhythmias, possibly due to an underlying cardiac condition. When relating patient comorbidities and predictions for the development of arrhythmias following inotropic administration, it is of value to mention that in the Dobutamine Compared to Milrinone (DOREMI) trial, it was found that ventricular arrhythmias were seen more in patients with chronic kidney disease, but it is not indisputable if this was due to milrinone toxicity, precisely [54].

Clinical trials demonstrating the favorable hemodynamic effects of augmented cardiac output and reduced ventricular pressures with the short-term administration of positive inotropic agents have previously led to the discovery of associated adverse effects.

Arrhythmias

The mechanism of action of the above two inotropes is known to contribute to the development of arrhythmias [50]. Due to their proarrhythmic potential, continuous monitoring of the cardiac rhythm is warranted during the intravenous administration of these drugs [40].

Because dobutamine lowers the atrial and atrioventricular (AV) node refractoriness and AV nodal conduction time along with boosting the sinoatrial automaticity, in humans, it has been shown to induce ventricular ectopic activity (VEA) in 3% to 15% of patients in clinical trials. However, the more severe and consequential VT has been seen rarely [50].

Intravenous milrinone has been found to likely cause arrhythmias in as many as 21% of the subjects on 48-hour milrinone infusions [40]. The common types of arrhythmias seen with the phosphodiesterase inhibitor class are sinus tachycardia and VEA, while supraventricular and ventricular arrhythmias have been reported rarely [50].

Tachycardia and Hypotension

In patients with heart failure, milrinone administration increases heart rate and frequently leads to a mild decrease in blood pressure. However, provided that the administration is limited to patients with high left ventricular filling pressure, clinically profound hypotension is not frequent. In a study conducted by Konstam and Cody [40], on the short-term use of intravenous milrinone for heart failure, clinically significant hypotension was seen in 2% of the subjects [40].

Infections

A pivotal retrospective study conducted by Acharya et al. [52] gave an idea of the development of adverse events in patients with stage D heart failure discharged on intravenous inotropes. Causes for subsequent hospitalizations included arrhythmias in 12% of patients, infections in 20% of patients, and worsening heart failure symptoms in 41% of the patients. Most of the subjects in this study faced at least one readmission after the initiation of inotropes. For the data collected on infection as the adverse effect, bacteremia was the most common type of infection. This adverse effect, however, can be attributed to the mode of administration of the long-term therapy, being a catheter or a central line [52].

Thrombocytopenia

In patients with heart failure on short-term intravenous milrinone, platelet counts have been reported to reach a minimum of 82,000/uL. It was seen in 2% of the subjects, and the cause of thrombocytopenia was attributed to milrinone in 1% [40].

Additional Reported Adverse Effects

Konstam and Cody [40] reported in their study the incidence of symptoms potentially attributable to myocardial ischemia in roughly 1% of the patients on short-term use of intravenous milrinone for heart failure. Headache was reported in 4% of the patients. [40].

Dobutamine and milrinone contraindications

Dobutamine and milrinone are not without side effects. Patients with severe pulmonary hypertension should be discouraged from being treated with milrinone since it could potentially increase vasodilation of pulmonary structures and, therefore, cause V/Q mismatch [55]. Milrinone is also contraindicated from being used as a long-term treatment for congestive heart failure (CHF); a prospective study found that daily use of oral milrinone increases morbidity and mortality in patients with CHF [56]. Patients with acute kidney injury or end-stage renal failure are also discouraged from being treated with milrinone. Milrinone is predominantly eliminated in urine (up to 90% of the drug), and therefore, impaired kidney function can increase the lifetime of milrinone in plasma [55,57]. A study comparing CHF outcomes when treated with milrinone suggested milrinone should be used carefully in patients with CHF of ischemic origin since it has worse consequences than acute decompensation of chronic CHF [34,58].

Dobutamine, on the other hand, has relatively lower contraindications. When administered the medication, people with severe hypertension can see an increase of up to 50 mmHg [59]. Hypokalemia is another situation in which there is a relative contraindication. A study reported that dobutamine produces a consistent decrease in plasma potassium [60,61]; therefore, potassium is administered simultaneously with dobutamine infusion in patients with hypokalemia.

Dobutamine and milrinone have been reported to interact with other cardiovascular drugs. Digoxin has been reported to increase the risk of arrhythmias in both of these drugs [51,41].

Clinical Manifestations of Arrhythmias

Although dobutamine is regarded as more pro-arrhythmogenic compared to milrinone, the occurrence and risk of morbidity and mortality due to drug-induced arrhythmias are comparable with the use of both medications [62]. Clinical manifestations of arrhythmias range from patient to patient. Supraventricular arrhythmias (usually atrial fibrillation/flutter) are the most common adverse events of vasopressor use at 45%, compared to 15% in ventricular arrhythmias [63].

Patients in CS are usually admitted to the ICU and are hemodynamically unstable. The presentation of arrhythmias may worsen the hemodynamic instability by worsening or causing tachycardia and tachypnea. Patients may also show signs of decreased/impaired responsiveness. Of note, limited data is available regarding the impact of arrhythmias caused by dobutamine and milrinone. It is reported that arrhythmias occur in 12% of individuals admitted to the ICU, with noted increased mortality in patients who experienced ventricular arrhythmias [64].

Ventricular arrhythmias also increase the risk of ST-elevation myocardial infarction (STEMI-CS), cardiac arrest, neurological sequelae, and mechanical ventilation use [65]. Supraventricular events were associated with an increased risk of renewed cardiac arrests and length of hospital stay [66]. Persons are also at increased risk of focal neurological events (stroke). Clinical trials are needed to determine the extent of the impact of arrhythmias caused by dobutamine and milrinone in CS.

Mortality on arrhythmias attributed to milrinone and dobutamine

Lewis et al. [13] found that arrhythmias have a statistically significant increase in patients treated with dobutamine compared to a group with milrinone [13]. Limited data was found regarding the mortality rate of arrhythmias when given either milrinone or dobutamine. A study evaluating the outcome of VT in patients treated with milrinone found that about 68% of the patients treated experienced more than three periods of sustained VT and had a mortality rate of 73% [67]. Supraventricular arrhythmias were not associated with an increased mortality risk [41]. Noted relative risk was 0.97; 95% CI of 0.68-1.40, and a p-value of 0.879. Ventricular events were also positively associated with increased mortality with a relative risk of 1.66; 95% CI 1.13-2.43; p = 0.026) [66]. In the absence of adequate papers on this topic, it is difficult to determine the exact mortality of patients with CS who were treated with milrinone and dobutamine and experienced arrhythmic events.

Pharmacological and non-pharmacological management strategies for arrhythmias

Pharmacological Management Strategies

**Figure 5:**
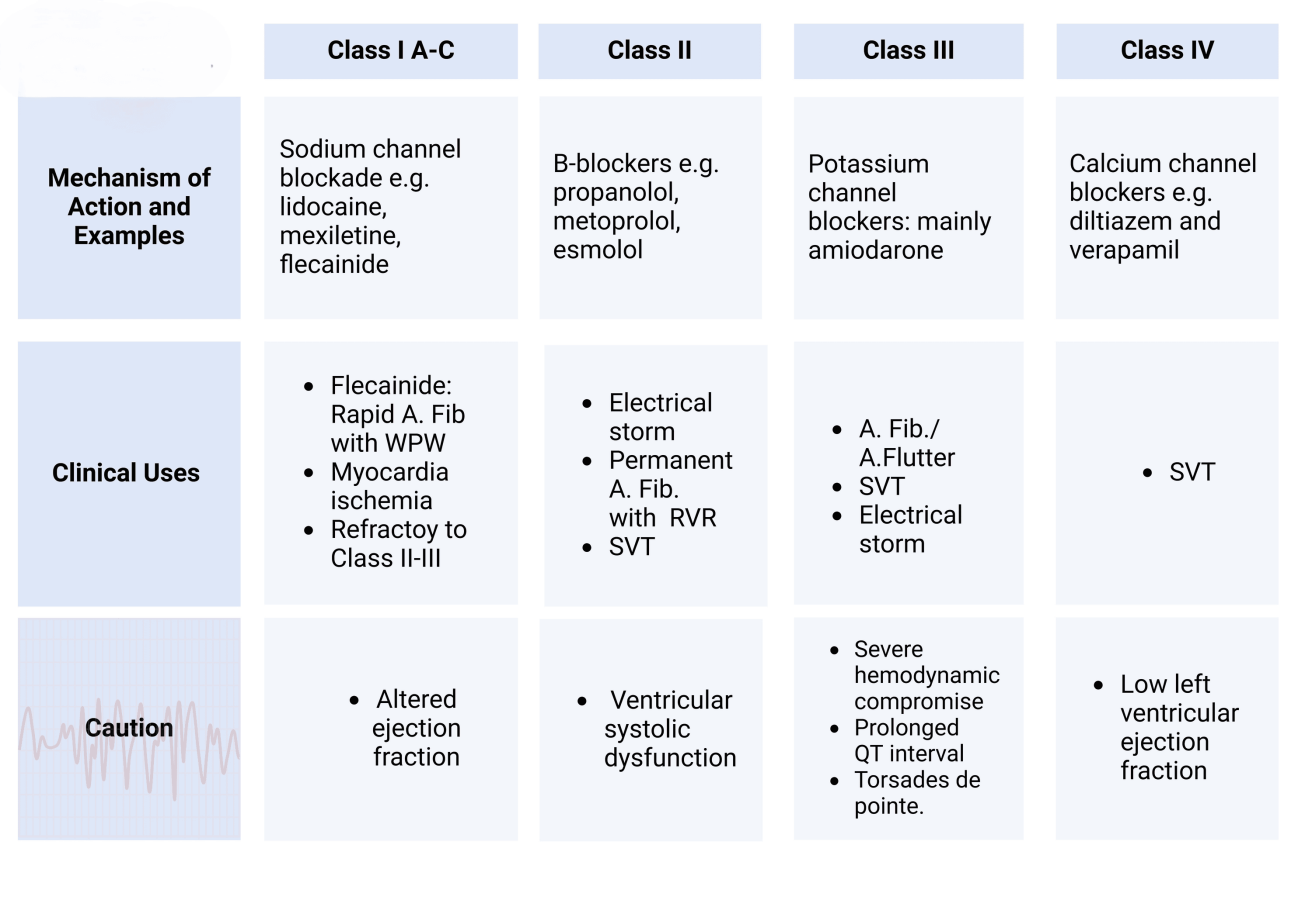
Anti arrhythmic medication used in cardiogenic shock Image Credits: Jodi-Ann A, Alexander Brooks WPW: Wolff-Parkinson-White syndrome; SVT: supraventricular tachycardia; RVR: rapid ventricular response

Laghlam et al. [68] noted that patients with CS have an increased prevalence of arrhythmias, including atrial fibrillation, VT, and AV nodal blocks [68]. Vallabhajosyula et al. [65], in a retrospective study of the arrhythmogenic burden seen in patients with CS-induced by myocardial infarction, deduced that up to 50% of the patients included had an arrhythmia and postulated the role of inotropes as a possible contributing factor [65]. Notably, the two main inotropes, dobutamine and milrinone, used in the treatment of CS, cause atrial and ventricular arrhythmias as a side effect. Milrinone may be administered with beta-blockers to prevent arrhythmias. In contrast, beta blockers are not used concomitantly with dobutamine [69]. Management of arrhythmias in CS involves early recognition of arrhythmias through monitoring, identification of triggers, treatment of the underlying cause, and treatment of arrhythmias through pharmacologic and/or non-pharmacologic means. Commonly utilized antiarrhythmic drugs are shown in Figure 5 [68].

Ventricular arrhythmias: Ventricular fibrillation caused by inotrope infusion is treated by discontinuing the drugs, especially dobutamine infusion, and performing cardiopulmonary resuscitation. In ventricular arrhythmias, administering intravenous β-blockers, for example, metoprolol at a 5-10 mg dose over five minutes, is indicated as a natural dobutamine antagonist. Intravenous procainamide (10 mg/kg body weight over five minutes) or amiodarone (150- to 300-mg bolus) can be given in β-blocker-resistant sustained VT [48,49].

Supraventricular arrhythmias: SVTs are most commonly associated with left ventricular dysfunction. Left ventricular dysfunction causes increasing pressures in the left atrium with an increase in size, predisposing to arrhythmias. While there are limited guidelines relating to the management of SVTs in CS, recent guidelines from the American Heart Association help guide treatment in acute atrial fibrillation. Atrial flutter is treated as per atrial fibrillation protocols. The goal is to restore sinus rhythm in those with hemodynamic compromise, and for those who are hemodynamically stable, either a rhythm or rate control strategy may be employed. Amiodarone is the drug of choice for cardioversion in critically ill patients with atrial fibrillation. One particular scenario is atrial fibrillation with Wolff-Parkinson-White syndrome, in which case flecainide may be used. Anticoagulation use is also to be guided by patients' CHA2DS2-VASC score and duration of atrial fibrillation, where recent studies indicate that a duration greater than 12 hours poses an increased thromboembolic risk [68]. Adenosine has a very short half-life, and therefore, adverse reactions like facial flushing and dyspnea last only for a short time. Amiodarone and digoxin may also be utilized. Where the SVT is refractory to pharmacologic drugs, there should be a low threshold for cardiac ablation therapy [70].

Non-pharmacological Management Strategies

For the patient who is hemodynamically unstable or refractory to antiarrhythmics, non-pharmacological interventions may be considered [71]. These include direct cardioversion or electrical defibrillation, in which DC electrical shock is applied to the heart. This electrical current depolarizes critical myocardial cells and interrupts disorganized ectopic impulses. It also allows the SA node to regain control as the dominant pacemaker. There are synchronized and non-synchronized DC cardioversion [72]. Effective against tachyarrhythmias due to reentrant (PSVTs, AF, VT, and VF). Other interventions include percutaneous right ventricular catheter ablation, which is minimally invasive and is effective for AVRT, AVNRT, atrial flutter, atrial fibrillation, and VT [71,73]. Cardiac ablation uses high-frequency energy or cryoablation to create scars. These scars will regulate the heartbeat. Electrophysiology study (EP study) uses sensor tips to find the origin of irregular beats, and then energy is applied to control irregular heartbeats [74]. Many show improvements after this procedure, and their quality of life improves. Another intervention used in emergent situations to control arrhythmias is temporary pacing. It is used to suppress the overdrive of atria to control recurrent ventricular arrhythmias. It can also be used as a bridge until a permanent pacemaker is implanted. The procedure has significant mortality rates and complications like hematoma, worsening of tachyarrhythmias, and sepsis [75].

Monitoring for early detection and management

Monitoring and treatment of sustained arrhythmias for patients with CS is critical to limit tachycardias that may worsen heart failure. Notably, the most common arrhythmia in patients with heart disease requiring intensive care is atrial fibrillation, which reduces left ventricular diastolic filling and lowers left ventricular ejection fraction, thereby worsening heart failure [70]. Early strategies for monitoring CS include non-invasive and invasive methods. Serial vital signs assessment, physical examination, continuous cardiac monitoring, serial echocardiography, and, less commonly, pulmonary artery catheterization (PAC) provide prognostic information [76]. One study showed that the resolution of lactatemia helped increase survival in patients with CS [68]. Using multiple modalities - serial echocardiography, biomarker assessment, peripheral arterial catheterization, and PAC - improves 30-day survival from 47% pre-intervention to 58% and 77% in the two subsequent years post-implementation [77].

Physicians assess stable or unstable patients via clinical examination and hemodynamic monitoring. Serial bedside observations include vital signs assessments, indicators of perfusion and cardiac function, namely, general assessment of the level of consciousness and respiratory effort, auscultation of lung and bowel sounds, evaluation of the peripheral vascular system through capillary refill time, edema, skin temperature, and monitoring of urinary output via an indwelling catheter. Peripheral arterial catheterization is recommended as a valuable adjunct for accurate and continuous assessment of patients' arterial pressures [77].

Serial serum chemistry evaluation helps diagnose CS and is valuable in assessing these patients' prognoses. Renal function testing, liver function testing, and serum lactate are valuable indicators of end-organ perfusion [77]. In terms of therapeutics, drug-specific monitoring is also helpful in patients receiving inotropes. In the setting of milrinone, liver function testing and platelet counts should be monitored due to possible adverse events. Given the risk of accumulation with renal dysfunction, urea, and creatinine may also reasonably be assessed during therapy. Electrolytes should be monitored with dobutamine use due to the risk of hypokalemia [78]. In patients with CS, the interval arterial value of lactate is predictive of mortality. The interval value at eight hours was found to be the best predictor of mortality in patients with acute myocardial infarction-induced CS, as compared to baseline values and the calculation of lactate clearance. A secondary analysis of patients with CS who had serum lactate measurements at 6, 12, and 24 hours found that values at these intervals were predictors of 30-day mortality [77]. In addition, dynamic changes in serum lactate levels in patients who are on extracorporeal life support (ECLS) have been predictive of mortality in patients with CS following myocardial infarction [79]. Furthermore, the relative change in lactate in the first 24 hours predicted survival [77]. Thus, serial analysis of renal and liver function tests and lactate levels are useful prognostic markers in CS but may also be indicated to monitor for toxicity with current therapeutic agents.

Echocardiography is a quick, non-invasive tool that may be used in the diagnosis and risk stratification of patients with CS, and it forms part of the basis for guiding therapeutic intervention. Assessment of ventricular wall contractility helps with the diagnosis of CS. With the evaluation of multiple parameters, a large retrospective database study showed that echocardiography correlates with SCAI stages and mortality rate, more so in patients with less severe stages of CS. In patients with atrial fibrillation complicating CS, transesophageal echocardiography guides the use of anticoagulation in patients requiring cardioversion [70]. The corrected left ventricular ejection fraction time ratio to pulmonary artery wedge pressure was shown to independently predict successful weaning from veno-arterial extracorporeal support [77]. Thus, echocardiography is fundamental in monitoring CS patients for diagnostic and therapeutic endpoints.

Though debatable at present, many clinicians in current practice, as well as expert recommendations, consider PAC for hemodynamic monitoring in patients with CS, particularly those with complicated presentations. Recently, in a multicenter retrospective review from the Cardiogenic Shock Working Group, stratifying patients based on the SCAI stage and degree of PAC use before the initiation of mechanical support, a statistically significant difference was reported in mortality rate based on PAC usage and SCAI stage. Furthermore, the lowest in-hospital mortality across all SCAI stages was noted in the patient group with complete PAC assessment. Specific data provided by PAC include diagnosing mixed shock, in which patients have a significant paraplegic component in addition to CS, as opposed to normotensive CS when there is a compensatory elevated vasomotor tone despite hypoperfusion due to cardiac dysfunction. PAC also helps to stratify patients as "wet vs. dry" and "warm vs. cold." The primary benefit of utilizing this classification is that it helps to guide the use of vasopressors, inotropes, and volume management. In addition, serial invasive monitoring and an aim to increase central venous oxygen saturation (ScvO_2_) may also be helpful. PAC may be beneficial in patients receiving mechanical circulatory support by guiding their withdrawal following recovery, or it may be necessary for adjunct pharmacologic support [68]. Overall, though no definitive guidelines exist, PAC usage in CS helps with diagnosing and stratifying types of CS for specific pharmacologic care, and with serial assessment, PAC helps to gauge patients' response to treatment to guide further care and possible need for the escalation of care [77].

Current limitations in management

Few relative contraindications have been described for dobutamine and milrinone. Idiopathic hypertrophic subaortic stenosis is one recognized contraindication to dobutamine, and some institutions also consider the past medical history of uncontrolled hypertension, recent myocardial infarction, large aortic aneurysm, and aortic dissections as well. Close monitoring and decreased doses of dobutamine are recommended in patients receiving monoamine oxidase inhibitors [78,79]. Due to increased conductance through the AV node in patients receiving dobutamine, caution should be employed in those prone to atrial fibrillation due to the risk of propagation into ventricular fibrillation. The chronotropic effect of dobutamine is dose-related, and at higher doses, tachycardia may mitigate the drug's beneficial effects on cardiac output. This risk can be reduced by adding low-dose dopamine rather than increasing the dose of dobutamine. Milrinone requires dose adjustment in the setting of renal dysfunction due to its renal clearance [80].

Individualized treatment based on risk factors, comorbidities, and pharmacogenomic aspects of both drugs

Pharmacogenomics is the branch of pharmacology that studies how varying drugs affect individuals based on their genetic profile [81]. An example of optimizing care of the population is that some studies show that there was a reduced response from milrinone among women and Black patients. Similarly, the ADRB1 389 polymorphism in women infused with dobutamine over two hours resulted in tachycardia and diastolic hypotension [82]. Risk stratification allows for individualized treatment strategies and guides the escalation of care by an expert team. In CS, acute cardiac dysfunction, as opposed to acute-on-chronic dysfunction, with its concomitant higher levels of lactate and a higher Sequential Organ Failure Assessment (SOFA) score, was shown to be associated with increased mortality [68]. A good predictor of overall survival was the risk assessment tool based on the National Cardiogenic Shock Initiative (NCSI) dataset, which comprises the cardiac power output and serum lactate values at 12-24 hours after shock presentation [79]. The managing team can consider these parameters to guide therapy.

Based on findings of the Dobutamine Compared with Milrinone (DOREMI) trial, the inodilators, dobutamine, and milrinone may be considered first-line agents for CS with preserved blood pressure, with dobutamine favored in the setting of renal impairment and in terms of cost savings for the total acquisition of the drug [83]. One significant adverse effect of these agents is cardiac arrhythmias, warranting close monitoring and opening the door to developing validated scores using clinical prognostic markers to risk-stratify patients for individualized management [84]. Currently, rescue agents of choice in CS are limited by their risks of individual toxicity. For the two drugs, milrinone and dobutamine, adverse effects reported leading to discontinuation rates are statistically the same but were more likely to be related to hypotension (13.1% vs. 0%, p < 0.01) in patients receiving milrinone and for arrhythmias in patients receiving dobutamine. Furthermore, in the case of milrinone, it is excreted by the kidneys, leading to the theoretically increased risk of adverse effects with renal dysfunction due to the prolongation of the half-life and its pharmacologic effects [85]. There exists no antidote or reversal agent, and thus, the mainstay of management in the setting of milrinone overdose is supportive management [51].

In contrast, dobutamine was found to be more likely to cause arrhythmias than milrinone (62.9% vs. 32.8%, p < 0.01), the difference primarily due to sinus tachycardia in patients on dobutamine [13]. This unrelenting tachycardia can be managed with a cardioselective beta-blocker such as metoprolol [41]. Therefore, managing adverse effects is typically limited to discontinuing the treatment and stabilizing the patient by alternate means, focusing on hemodynamic parameters.

Potential future treatment options

Given the proarrhythmic nature of CS, risk stratification to guide therapeutic strategies such as preventive antiarrhythmics is a novel area of research with no validated scoring systems at present. However, based on a randomized control trial, it was shown that several clinical parameters are associated with the risk of arrhythmias in CS. In CS, there is a positive correlation between the use of a vasopressor upon initiation of an inotrope or a history of atrial fibrillation and the development of arrhythmias. There was a negative association between increasing left ventricular ejection fraction, a previous myocardial infarction, and predominant right ventricular dysfunction [66]. While not currently validated, these observations may guide clinical management regarding a lower threshold to treatment escalation and adjuncts such as mechanical circulatory support and definitive cardiac transplantation. Risk stratification tools and algorithms are areas for future research.

Future initiatives should include robust, randomized, double-blind clinical trials to guide definitive clinical decision-making. Currently, data is lacking comparing all causes of mortality between milrinone and dobutamine in CS [86]. However, to date, it has been shown that compared to a placebo, it was uncertain whether milrinone (OR: 0.52; 95% CI, 0.19 to 1.39) or dobutamine reduced mortality as compared to a placebo (OR: 0.67; 95% CI, 0.30 to 1.49). However, another indicator, levosimendan, a calcium sanitizer, was shown to possibly be more favorable in the subset of patients with lower severity of the CS, as compared to a placebo (OR: 0.53; 95% CI, 0.33 to 0.87) [87].

Levosimendan has similar indications to dobutamine and milrinone, CS with preserved blood pressure, but has been shown to have a reduced risk of arrhythmias [36]. Levosimendan performed better than dobutamine in maintaining the patient's hemodynamic stability and was better regarding myocardial efficiency [88]. Levosimendan required less myocardial oxygen consumption than milrinone to improve cardiac contraction similarly. Another study highlighted that this drug avoids activating adrenergic pathways and increases contractility without raising calcium levels. This avoids higher energy consumption through the activation of calcium-transporting systems; hence, levosimendan may be associated with fewer arrhythmias than conventional inotropes [89]. Despite the advantageous profile of this medication, it has a considerable risk of hypotension in CS and a prolonged time of onset, 2-5 hours [90]. Further trials are needed to compare these three inotropes in CS.

## Conclusions

Our study assesses the effect of milrinone and dobutamine, which are used for CS. Various studies concluded that both drugs cause significant atrial and ventricular arrhythmias that increase rehospitalization and mortality. The risk of arrhythmias due to these drugs can be reduced by decreasing the dose and duration of use and avoiding it in patients with comorbidities. Future studies are required to analyze the various causes of mortality of these drugs in CS and determine the effect of levosimendan, which consumes less oxygen and has fewer arrhythmogenic effects compared to the other two inotropes.
